# Addressing Incidental Findings in Neuroimaging Studies: Never Easy, Rarely Rescue

**DOI:** 10.1002/eahr.70006

**Published:** 2026-03-05

**Authors:** Hayden P. Nix

**Affiliations:** ^1^ Internal medicine resident physician in the Department of Medicine at Dalhousie University in Halifax

**Keywords:** human subjects research, medical imaging research, brain scans, informed consent, duty to disclose, ancillary care obligations

## Abstract

Population‐wide neuroimaging studies reveal brain lesions in approximately 3% of putatively healthy research participants. It is unclear how researchers ought to address this problem and whether they should seek out incidental findings. Koplin and colleagues argue that researchers have a duty of easy rescue and therefore a duty to look for and disclose potentially serious incidental findings to research participants in neuroimaging studies. In this article, I argue that the duty of easy rescue is an inappropriate approach to this ethical problem. The duty of easy rescue paradigmatically applies to cases in which the intervention is (1) minimally burdensome to the rescuer, (2) virtually certain to minimally burden the individual in danger, and (3) virtually certain to confer benefit to the individual in danger. By contrast, looking for and disclosing potentially serious incidental findings is (1) often burdensome to researchers, (2) poses the risk of burdening research participants with false positive findings, and (3) even when a potentially serious incidental finding is found, disclosed, and medically managed, doing so may not confer benefit to the research participant. The duty of easy rescue is not a useful tool for navigating the ethical problems raised by incidental findings in population‐wide neuroimaging research.

Recently, there has been a boom in medical imaging research. Population‐wide cohort studies, including the UK Biobank Imaging Study, the German National Cohort Magnetic Resonance Imaging Study, and the Brain Research through Advancing Innovative Neurotechnologies Initiative in the United States have compiled databases of tens of thousands of brain scans taken from putatively healthy research participants. This area of research aims to elucidate the determinants of a range of diseases to improve their prevention, diagnosis, and treatment. An estimated 3% of these scans yield an incidental finding,^1^ which is defined as “a finding concerning an individual research participant that has potential health or reproductive importance and is discovered in the course of conducting research but is beyond the aims of this study.”^2(p.219)^

Incidental findings raise ethical questions about researchers’ obligations to research participants. There is broad consensus that researchers should establish protocols for the management of incidental findings likely to be discovered in their specific study, and that the protocol should be disclosed to potential research participants during informed consent discussions.^3^, ^4^ Further, there is growing consensus that researchers have a duty to disclose clinically significant incidental findings to research participants.^5^, ^6^, ^7^, ^8^

However, there is ongoing debate about the ethical foundation for the duty to disclose clinically significant incidental findings. Miller and colleagues argue that this duty is grounded in the professional relationship between researchers and research participants.^5^ Within the relationship, research participants are vulnerable and researchers are in a privileged position to access private information; if researchers have the competence to identify clinically significant incidental findings, then they have an obligation to disclose them to research participants. Rangel argues that the duty to disclose clinically significant incidental findings is grounded in researchers’ ancillary care obligations; research participants’ partial entrustment of health‐related interests in researchers creates a duty for researchers to protect those interests.^7^ Graham and colleagues argue that the duty is grounded in researchers’ distributive justice obligations; publicly funded researchers, as agents of the state, have obligations to provide basic care, and therefore a duty to disclose clinically significant incidental findings to research participants.^8^ Koplin and colleagues appeal to a more basic ethical principle. They argue that the duty to disclose clinically significant incidental findings is grounded in the duty of easy rescue.^6^

Koplin and colleagues take their argument one step further by arguing that, in addition to the duty to disclose clinically significant incidental findings, the duty of easy rescue grounds an ethical obligation for researchers to actively seek out potentially clinically significant incidental findings.^6^ This argument is controversial. The President's Commission for the Study of Bioethical Issues recommends that researchers do not have an obligation to look for clinically significant findings that are outside the study objective.^3^ Similarly, leading scholars in the field of incidental findings argue that looking for incidental findings falls outside of researchers’ obligations.^5^, ^9^

In this article, I criticize Koplin and colleagues’ position. There may be good reasons for researchers to look for and disclose potentially serious incidental findings to research participants in population‐wide neuroimaging studies. However, I argue that the duty of easy rescue does not ground a duty for researchers to do so. The article progresses as follows: First, I explore the duty of easy rescue, as recapitulated by Koplin and colleagues, and identify key features of easy rescues. Second, I summarize Koplin and colleagues’ argument that the duty of easy rescue grounds a duty to look for and disclose potentially serious incidental findings in neuroimaging research. Third, I argue that the duty of easy rescue is not a useful tool to help researchers navigate the ethical problems raised by incidental findings in population‐wide neuroimaging research because (1) the resources required to reduce the risk of false positive findings would be burdensome to researchers; (2) looking for and disclosing potentially serious incidental findings poses the risk of burdening research participants with false positives; and (3) even when a serious incidental finding is sought, accurately identified, disclosed, and medically managed, doing so may not confer benefit to the research participant. I conclude that the duty of easy rescue does not meaningfully guide researchers faced with problems posed by incidental findings in population‐wide neuroimaging research.

## DUTY OF EASY RESCUE

Koplin and colleagues explicate the duty of easy rescue with reference to seminal works by Singer and Bentham. Singer argues that every moral agent has a duty of easy rescue.^10^ According to Singer, “if it is in our power to prevent something bad from happening,

**The duty of easy rescue is not a useful tool to help researchers navigate the ethical problems raised by incidental findings in population‐wide neuroimaging research.**

without thereby sacrificing anything of comparable moral importance, we ought, morally, to do it.”^10(p.231)^ To explicate this principle, Singer invokes a simple thought experiment: “If I am walking past a shallow pond and see a child drowning in it, I ought to wade in and pull the child out. This will mean getting my clothes muddy, but this is insignificant, while the death of the child would presumably be a very bad thing.”^10(p.231)^

Other thought experiments have also been used to explicate the duty of easy rescue. Bentham provides three widely accepted examples of cases in which the bystander has a clear duty of easy rescue: “A woman's head‐dress catches fire: water is at hand: a man, instead of assisting to quench the fire, looks on, and laughs at it. A drunken man, falling with his face downwards into a puddle, is in danger of suffocation: lifting his head a little on one side would save him: another man sees this and lets him lie. A quantity of gunpowder lies scattered about a room: a man is going into it with a lighted candle: another, knowing this, lets him go in without warning.”^11(p.55)^

These examples demonstrate the kinds of interventions to which the duty of easy rescue applies. There are three key features of note. First, it must be virtually certain that intervening will minimally burden the rescuer. In each thought experiment, the rescue involves only a simple task. Wading into a pond, extinguishing a fire with water at hand, helping someone turn their head, and issuing a verbal warning are not burdensome for the rescuer. As Singer argues, if the burden on the rescuer is too great, the duty of easy rescue does not apply. Second, it must be virtually certain that the intervention will minimally burden the individual in danger. Pulling a child to shore, pouring water on an individual's hair, lifting an individual's head, and issuing a verbal warning do not pose any substantial risk to the recipient of the intervention. And third, it must be virtually certain that the intervention will benefit the individual in danger. In each thought experiment, the bystander has the means to perform the intervention. The passerby at the shallow pond and the man next to the person who is drunk are abled‐bodied; the man next to the woman on fire has water at hand; and the man next to the gunpowder room possesses knowledge of the room's contents and the means to warn the candleholder. In each case, if the bystander chooses to intervene, it is virtually certain that the individual in danger would be saved. These three features: minimal risk of burden to rescuer, minimal risk of burden to individual in danger, and virtual certainty of benefit to individual in danger, are necessary conditions for the duty of easy rescue to apply to a situation.

## DUTY TO LOOK FOR AND DISCLOSE POTENTIALLY SERIOUS INCIDENTAL FINDINGS

Koplin and colleagues argue that the duty of easy rescue grounds a duty for researchers to look for and disclose potentially serious incidental findings in population‐wide neuroimaging studies of healthy volunteers. They argue that the researchers conducting these studies, like all moral agents, have a duty to perform easy rescues. They endorse a duty of rescue with a moderate strength, meaning a duty of easy rescue that “is not limited to *imminent* risks of extreme *harm*; instead, it applies to the broader category of *harms that are large relative to the cost of preventing them* (and where the cost of preventing this harm is reasonably bearable).”^6(p.6)^ They conceptualize two interventions that may constitute easy rescues: (1) looking for serious incidental findings, and (2) disclosing serious incidental findings to research participants and providing subsequent medical care.

Koplin and colleagues specify two limitations on the scope of the duty to look for and disclose potentially serious incidental findings. The first limitation is that the duty only applies to incidental findings of high clinical validity and utility. While Koplin and colleagues do not define clinical validity and utility, validity can be understood as “the accuracy and reliability of the finding,” and utility can be understood as “the potential health or reproductive importance of the findings to the patient.”^8(270)^ This limitation makes clear that if looking for and disclosing an incidental finding is unlikely to effectively promote the participant's well‐being, then researchers do not have a duty to do so. This is consistent with my analysis of the duty of easy rescue, because the duty of easy rescue only applies to interventions that are virtually certain to benefit the individual in danger. While Koplin and colleagues do not specify which lesions count as potentially serious, they claim that the list developed by researchers conducting the UK Biobank Imaging Study provides “a useful test case” for their argument.^6^ The list includes acute brain infarction, acute hydrocephalus, acute intracranial hemorrhage, arachnoid cyst, colloid cyst of third ventricle, intracranial mass lesion, mastoiditis, and suspected intracranial aneurysm or vascular malformation.^12^ For the purpose of my argument, I will also use this list of potentially serious lesions as a test case.

Koplin and colleagues argue that the second limitation on the scope of the duty to look for and disclose potentially serious incidental findings is that if looking for and disclosing potentially serious incidental findings imposes too great a burden on researchers, then the duty is overridden and they do not have a duty to do so. This is because the duty of easy rescue does not apply to cases in which the burden on the rescuer is substantial.

## NOT EASY: RESEARCHER BURDEN

The duty of easy rescue is not an appropriate approach for the problem of incidental findings in population‐wide neuroimaging research because identifying clinically important incidental findings is difficult, and the resources required to enable researchers to do this with a high degree of specificity would be burdensome to researchers.

In population‐wide neuroimaging studies, researchers may employ radiographers, radiologists, or both to interpret the images. First, consider population‐wide neuroimaging studies in which radiographers are responsible for interpreting images. In these studies, looking for and disclosing a potentially serious incidental finding would rarely constitute an easy rescue because, generally, radiographers do not have sufficient training for this complex task. In clinical settings, radiographers are responsible for taking images of patients using various imaging technologies (e.g., X‐ray, computed tomography scans, and magnetic resonance imaging). They are not responsible for—or permitted to—interpret images to formulate a diagnosis because they do not possess the needed expertise. In population‐wide imaging studies, radiographers receive specific training to identify the lesion(s) of interest, according to the study's research question. Radiographers also often receive some training on identifying images for potentially serious lesions so that they can send them to radiologists. However, the training is insufficient for them to be able to reliably identify potentially serious lesions. One study by Gibson and colleagues had radiographers screen 1,000 neuroimages.^12^ Out of the 1,000 neuroimages, 21 contained a potentially serious lesion. The radiographers flagged 66 scans as having potentially serious lesions. Of these 66 scans, only 5 contained potentially serious lesions. This means that radiographer flagging resulted in 61 false positives and 16 false negatives. Their ability to identify potentially serious lesions was neither sensitive (24%) nor specific (6%).

Second, consider population‐wide neuroimaging studies in which radiologists are responsible for interpreting images. Radiologists undergo years of rigorous training to learn how to interpret medical images. Intuitively, it seems likely that radiologists’ expertise would allow them to reliably identify potentially serious lesions on neuroimages. In fact, Gibson and colleagues found that systematic radiologist review resulted in the correct identification of all 21 potentially serious lesions in the sample of 1,000 scans.^12^ However, systematic radiologist review also resulted in 158 false positives. This means that systematic radiologist review had high sensitivity (100%), but poor specificity (19%). In population‐wide neuroimaging studies, radiologists often do not have access to high‐resolution scans, participants’ clinical histories, or additional diagnostic investigations. These factors may have contributed to the low specificity seen in the study by Gibson and colleagues.

In population‐wide neuroimaging studies, research ethics regulatory bodies face a policy‐level dilemma: should researchers be required to look for and disclose potentially serious incidental findings? The empirical evidence presented above indicates that radiographers and radiologists are unable to identify potentially serious incidental findings on neuroimages with a high degree of specificity. High‐resolution imaging and the collection of clinical histories would likely reduce the frequency of false positives and increase specificity. However, the financial cost of employing radiologists, collecting clinical histories, and taking high‐resolution neuroimages would be infeasibly high for most studies. Koplin and colleagues concede that “[r]esearchers and research institutions do not have a moral duty to look for incidental findings if the costs of doing so are prohibitive or highly burdensome.”^6(p.9)^ Indeed, the duty of easy rescue only applies to situations in which intervening is minimally burdensome to the rescuer. Therefore, the duty of easy rescue does not ground a duty to look for and disclose potentially serious incidental findings in population‐wide neuroimaging studies because the resources required to do so with a high degree of specificity would be too burdensome on researchers.

## RARELY RESCUE: RESEARCH PARTICIPANT BURDEN

Whether images are interpreted by radiographers or radiologists, there are likely to be some cases in which looking for and disclosing potentially serious incidental findings constitutes an easy rescue in retrospect. For example, if a radiologist or an especially experienced radiographer correctly identifies a lesion on a neuroimage as a meningioma and the patient is informed and receives a total surgical resection, the act of looking for, disclosing, and medically managing the incidental finding could—in retrospect—be conceived of as an easy rescue.

However, looking for and disclosing a potentially serious incidental finding that ends up being a false positive does not constitute an easy rescue because it burdens the research participant. When a research participant is informed about a potentially serious incidental finding, they must undergo a series of investigations to determine its clinical significance. Koplin and colleagues concede that “false positives caused needless anxiety and unnecessary clinical assessment, which had negative impacts on some participants’ finances, access to insurance, and emotional well‐being.”^6^ In false positive cases, the research participant does not require medical treatment for the lesion and therefore derives little to no benefit from learning about the incidental finding. But the duty of easy rescue only applies to situations in which the intervention is virtually certain to minimally burden the individual in danger. Therefore, the duty of easy rescue is inapplicable to cases of false positive incidental findings.

The inapplicability of the duty of easy rescue to false positive cases renders the duty of easy rescue unhelpful for navigating the ethical problems raised by incidental findings in neuroimaging studies, in general. When a researcher goes looking for potentially serious incidental findings and when a researcher identifies a potentially serious incidental finding, they cannot know in advance whether it will turn out to be a false positive. Recall, the duty of easy rescue only applies to situations in which the intervention is virtually certain to minimally burden the individual in danger. The uncertainty surrounding whether looking for and disclosing incidental findings will ultimately burden research participants with false positive findings renders the duty of easy rescue inapplicable. Therefore, the duty of easy rescue is not a useful tool to help researchers navigate the ethical problems raised by incidental findings in population‐wide neuroimaging research.

## RARELY RESCUE: UNCERTAIN RESEARCH PARTICIPANT BENEFIT

Even when radiographers or radiologists correctly identify a serious incidental finding, the duty of easy rescue does not always apply. Within the UK Biobank Imaging Study's limited list of potentially serious lesions, the medical management participants receive for serious incidental findings often causes more burden than benefit, rendering the duty of easy rescue inapplicable.

As discussed above, there are some serious lesions that require immediate medical care. For example, identifying and informing research participants about acute brain infarcts, acute hydrocephalus, or brain tumors found on their neuroimages may confer benefit to them, because there are evidence‐based treatments for these lesions. In these cases, looking for, disclosing, and providing medical management for incidental findings may constitute an easy rescue.

However, for other lesions on the UK Biobank Imaging Study's list of potentially serious lesions, the standard‐of‐care medical management for the lesion is surveillance. For example, the standard of care for asymptomatic colloid cysts of the third ventricle is to monitor the patient with serial follow‐up imaging. In some cases, the cyst grows slowly and eventually blocks the flow of cerebrospinal fluid; these patients require neurosurgery to remove the cyst. However, in most cases the cyst does not grow and no treatment is needed. One study found that after ten years of follow‐up, 93 out of 98 patients with asymptomatic colloid cysts in the third ventricle did not need surgery.^13^ In cases where the standard‐of‐care management is surveillance, patients often continue to feel psychologically distressed after discussing the management plan with physicians.^14^ This distress often causes patients to pressure physicians to order additional diagnostic investigations to be sure that the incidental finding does not require an active intervention, which may strain the patient‐physician relationship.^14^ In these cases, the balance between the burdens and benefits conferred to the patient by looking for and disclosing the incidental finding is unclear. But the duty of easy rescue only applies to situations in which the intervention is virtually certain to benefit the individual in danger. Therefore, the duty of easy rescue is inapplicable to these cases.

Further, for other lesions on the UK Biobank Imaging Study's list of potentially serious incidental findings, physicians are uncertain how to treat patients because there is a paucity of evidence to guide clinical decision‐making.^14^ For example, neurologists and neurosurgeons are unsure whether incidentally found arterial venous malformations should be managed with surveillance or surgery.^14^ In these cases, physicians simply do not know how to promote the medical interests of patients. The duty of easy rescue is inapplicable in these cases, because these treatment decisions are made through a complex process of weighing the benefits and burdens of different interventions.

Using the lesions included on the list of potentially serious incidental findings identified by the UK Biobank Imaging Study as a test case reveals that looking for and disclosing incidental findings constitutes an easy rescue in a limited range of cases. In response to this argument, Koplin and colleagues might argue that the duty to look for and disclose incidental findings simply does not apply to these cases, and that they ought to be removed from the list of lesions that researchers should look for and disclose. However, this response is unsatisfactory because some patients with these lesions do benefit from medical care. For example, if a patient's colloid cyst of the third ventricle progresses, they are likely to benefit from surgery. This is a strong justification for keeping colloid cysts of the third ventricle on the list of mandatory disclosures, even though it would be inaccurate to characterize looking for them as an easy rescue. Therefore, modelling the issue of potentially serious incidental findings in terms of easy rescue is inappropriate. This issue requires nuanced analysis of trade‐offs between the burdens and benefits conferred to research participants, making the duty of easy rescue the wrong tool for the job.

## CONCLUSION

The duty of easy rescue is an inappropriate approach to the problem of how researchers ought to deal with looking for and and disclosing incidental findings in population‐wide neuroimaging studies. The duty of easy rescue only applies to cases in which the intervention is (1) minimally burdensome to the rescuer, (2) minimally burdensome to the individual in danger, and (3) virtually certain to benefit the individual in danger. By contrast, (1) looking for clinically important incidental findings with a high degree of specificity is burdensome to researchers; (2) looking for and disclosing potentially serious incidental findings poses the risk of burdening research participants with false positive findings; and (3) even when a potentially serious incidental finding is sought, accurately identified, disclosed, and medically managed, it is often uncertain whether this will confer benefit to the research participant. Deciding whether to look for and disclose potentially serious incidental findings in population‐wide neuroimaging studies requires nuanced analysis of risks and benefits. But the duty of easy rescue only applies to situations in which the benefits of an intervention clearly and substantially outweigh the risks. Therefore, the duty of easy rescue does not provide meaningful guidance to researchers faced with the problems posed by incidental findings in population‐wide neuroimaging research.

## ACKNOWLEDGMENT

The author is grateful for helpful comments provided by Cesar Palacios‐Gonzalez, Nicholas Murphy, and Charles Weijer.
